# Age Differences in the Influence of Residential Environment and Behavior on the Life Quality of Older Adults: The Transfer from Physical-Environment to Social-Behavior

**DOI:** 10.3390/ijerph18030895

**Published:** 2021-01-21

**Authors:** Zhenhua Zheng, Hong Chen, Junling Gao

**Affiliations:** 1 College of Communication and Art Design, University of Shanghai for Science and Technology, No. 516, Jungong Road, Shanghai 200093, China; 19527@tongji.edu.cn; 2 College of Architecture & Environment, Sichuan University, No. 24, First South Section, First Ring Road, Chengdu 610065, China; 3 School of Public Health, Fudan University, Shanghai 200032, China; jlgao@fudan.edu.cn

**Keywords:** residential environment, neighborhood interaction, outdoor exercise, age groups, the life quality of older adults

## Abstract

With the development of the concept of “ageing-friendly communities”, increasing attention has been paid to the effect of residential environments on the life quality of older adults. However, the logical relationship between residential environment, individual behavior and life quality of older adults has not been clearly revealed. Based on data in Shanghai, China, this study explored the relationships between residential environments and the life quality of older adults in different age groups, and analyzed the mediating role of individual behaviors (neighborhood interaction and outdoor exercise). The findings confirmed that residential environment, neighborhood interactions and outdoor exercise have significant positive effects on the life quality of older adults. Meanwhile, the impact of residential environment on the life quality of older adults is exclusively realized through the mediating role of individual behavior. However, there were significant differences in the model paths among various age groups. With ageing, the positive effects of residential environment on the quality of life gradually weakened, while that of neighborhood interaction gradually improved. The findings prove that the influencing factors on the quality of life of older adults tend to shift from residential environment to neighborhood interaction as the age of residents advances. This knowledge is crucial with regard to the differentiated and accurate design of older communities.

## 1. Introduction

The ageing of the population is becoming an important issue of concern for countries around the world. By 2050, the number of people aged 60 and above is expected to reach 2.5 billion, that is, about 21.3% of the world’s population [1]. As the pace of global ageing increases, improving the life quality of older adults has become an issue of significant concern as well as an important goal for governments. As the residential environment of the community is the primary activity site for older adults, an appropriate residential environment has an important bearing on the life quality of older adults. With the development of the concept, the theory, and the practice of “ageing-friendly communities”, a large number of studies have focused on the relationship between the residential environment and the health of older adults [2,3,4,5,6,7,8]. Meanwhile, more and more scholars are paying attention to the relationship between the residential environment and the life quality of older adults. Researchers such as Parra et al., Sirgy et al., Forjaz et al., Williams et al., Eyles and Williams, etc. used data from different countries to prove that the community environment has an important impact on the life quality of older adults [9,10,11,12,13,14]. 

In 1973, Lawton and Nahemow proposed the concept of an Ecological Model of Ageing [15]. The concept emphasizes the interaction among individuals, the physical environment, the behavioral mode and the well-being of older adults [6]. In the decades that followed, many scholars, based on the theory of the social-ecological model of older adults, discussed how the living environment affected the health of older adults by influencing their behavior [16,17,18,19,20,21]. However, the complex logic relationship among the residential environment, the behavior of older adults and the life quality of older adults remains to be revealed.

China is the country with the largest ageing population in the world. By the end of 2018, there were 249.49 million Chinese aged 60 and above, accounting for 17.9% of the total population [22]. At the same time, China is experiencing a rapid urbanization process. Therefore, studying the relationship among the residential environment, the behavior of individuals and the life quality of older adults in urban China is vitally important for improving the life quality of both older adults and Chinese people as a whole. 

The present study is based on the social-ecological model of older adults and the statistical method of the structural equation model, respectively. This study uses survey data from Shanghai. Our research analyzed how the residential environment affects the life quality of older adults by influencing their individual behaviors, and compared the differences between different age groups of older adults. The research findings will help to provide effective support for the construction of ageing-friendly communities and the accurate management of residential districts.

## 2. Construction of the Social–Ecological Model of “Residential Environment–Individual Behavior–Life Quality Relationship of Older Adults”

**Figure 1:**
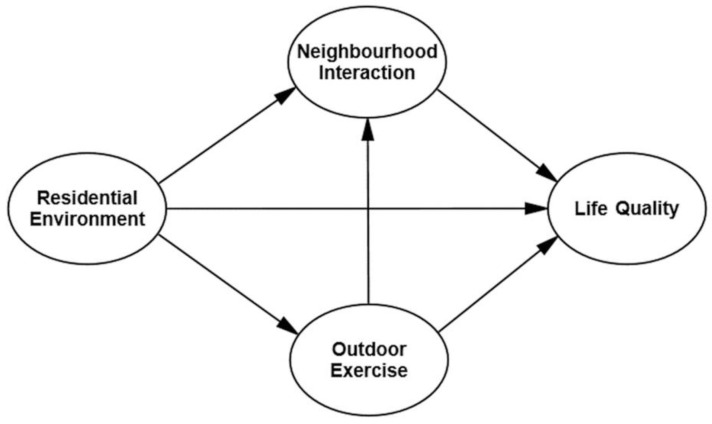
Social–ecological model of “residential environment–individual behavior–life quality relationship of older adults”.

For older adults who live at home, most of their daily activities are carried out in their residential environments. Among the activities, outdoor exercise (personal behavior) and interaction with neighbors (social behavior) are considered to be the most important individual behavior modes that influence the health and well-being of older adults [23,24,25,26,27]. Therefore, the social ecosystem of older adults’ residential area is composed of many factors including the older adults themselves, the residential environment, the individual behavior (including outdoor exercise, neighborhood interaction) and the well-being of the older adults. It not only emphasizes the important role of the residential environment on the well-being of older adults, but it equally emphasizes the joint role of the individual behavior of older adults in this ecosystem [3,6]. Drawing from the above analyses, it is our assumption that the impact of the residential environment on the life quality of older adults does not exist in isolation. Rather, it exists through the intermediary role of neighborhood interactions and outdoor exercise. Therefore, we constructed a social–ecological model of “residential environment–individual behavior–life quality relationship of older adults”, as shown in Figure 1.

## 3. Methods and Measures

### 3.1. Study Design

The area selected for the current study is Xinhua Street in Changning District, located in the downtown area of Shanghai, China. The data were collected from a large household survey sample conducted in 2014. The subjects of this survey were older adults aged 60 and above and who resided in communities. The purpose of the survey was to investigate the relationship among the residential environment, outdoor exercise, neighborhood interaction and the life quality of older adults. 

### 3.2. Study Participants and Procedures

Xinhua Street is located in Changning District, Shanghai. The street comprises 17 residential areas and 198 residential districts that cover an area of approximately 2.2 km^2^. The street has a population of about 78,000 inhabitants. A two-stage sampling method was employed in the survey: first, the residential district samples were selected and, second, the older adult samples were selected. 

**Figure 2:**
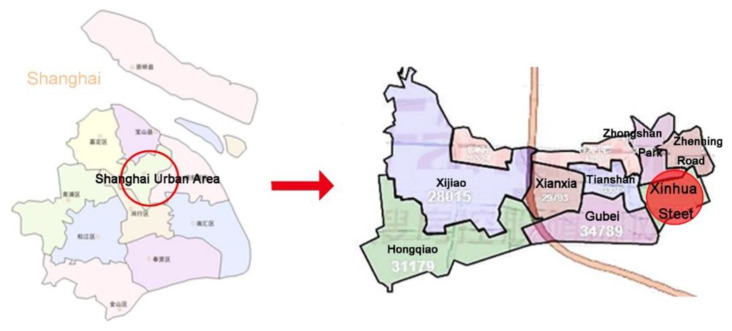
Location of Xinhua Street.

**Figure 3:**
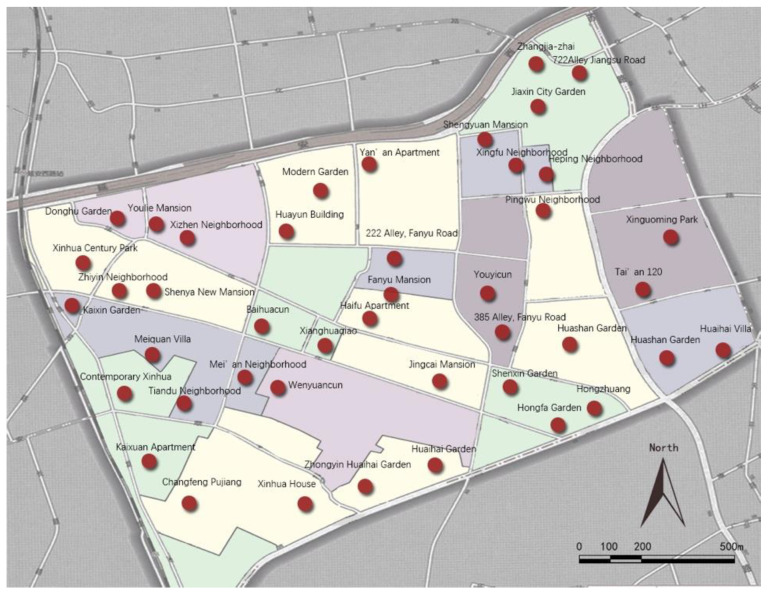
Map of the residential districts sample.

The representativity of the sampling principles for residential districts was enhanced in a number of ways: (1) The equilibrium of the geographical location. The selection of residential district samples was, as much as possible, aimed at achieving a balanced geographical distribution within Xinhua Street. (2) The diversity in the years of construction of the residential districts. Samples of selected residential districts built between the 1980s to the 2000s. (3) The diversity of housing types. The samples covered as many housing types as possible, including, for example, low-rise, multi-storey, and high-rise buildings, as well as brick–concrete, reinforced concrete, and steel structures. Finally, as shown in Figure 2 and Figure 3, a total of 43 residential districts were selected from 198 residential districts in Xinhua Street. 

In the second stage, in the selected residential districts, older adults aged 60 and above who voluntarily participated in the survey without cognitive impairment were selected as the survey objects. If the number of eligible older adults in the selected community was less than 120, all were surveyed. However, if the number of eligible older adults in the selected community was over 120, then a simple random sampling method was adopted in the selection of the 120 older adults to survey. Excluding the invalid samples, the final number of valid samples was 2783. 

To compare the results among various ages, all valid samples were divided into 3 groups based on the ages of participants. The low-age group was 60–69; the middle-age group was 70–79; the high-age group was 80 and above. The total number of samples was 1292 for the low-age group, 964 samples for the middle-age group, and 527 samples for the high-age group.

### 3.3. Measures

#### 3.3.1. Dependent Variable: Life Quality of Older Adults

The concept of "quality of life" represents the degree to which human needs are met, or the degree to which individuals or groups of individuals are satisfied or dissatisfied with all aspects of life [28,29,30,31]. The measurement of the quality of life includes objective and subjective measurements. 

Although the measurement of objective quality of life may reflect how certain material and social needs are met, it is incomplete and it lacks data on important psychological feelings. In fact, many objective measures are based on the subjective experience of the decision maker [31]. However, the fundamental goal of the subjective quality of life is the overall development and well-being promotion of individuals that equally reflects the ultimate concern for people. The subjective quality of life better reflects the degree to which individual needs are met [32]. Therefore, currently, an increasing number of scholars are beginning to evaluate the subjective quality of life [33,34,35].

Based on the above, this paper uses the subjective quality of life as an indicator to measure the quality of life. In particular, it measures the subjective feelings of older adults on their overall living conditions. The life quality of older adults was assessed based on their responses to 8 questions. Respondents were asked to rate their quality of life status on a scale of 1 to 10, where 1 signified a very low life quality while 10 represented a very high life quality. 

#### 3.3.2. Independent Variable: Residential Environment

In this paper, the residential environment is based on two measurement scales of the settlement-aware environment developed by Mujahid et al. [36]. It is defined as the outdoor physical environment provided by the community, including the leisure environment and the landscape environment. The responses to each item ranged from 1 to 5 (1 = completely disagree, 2 = disagree, 3 = neutral, 4 = completely agree, 5 = agree). A higher score indicated a higher degree of acceptance of their residential environment by older adults. The variables description and sample conditions are shown in Table 1.

#### 3.3.3. Intermediary Variables: Outdoor Exercise and Neighborhood Interaction

The mediating variables included two aspects of individual behavior of older adults: outdoor exercise that reflects personal behavior and neighborhood interaction which reflects social behavior. Outdoor exercise comprised two observation variables: walking frequency and walking duration. Neighborhood interaction included five indicators: help, care, activities, communication, and chatting. Each indicator had a score range from 1 to 4 (1 = never, 2 = occasional, 3 = sometimes, 4 = often), representing the frequency of neighborhood interaction.

#### 3.3.4. Control Variable

The socioeconomic status and the length of residence were selected as control variables in this paper. Socioeconomic status included income and education. Participants were requested to choose their level of income on scale from 1 to 6 (1 = <1500 yuan, 2 = 1500–2500 yuan, 3 = 2500–3500 yuan, 4 = 3500–4500 yuan, 5 = 4500–5500 yuan, 6 = >5500 yuan). The score of education ranged from 1 to 6 (1 = junior high school and below, 2 = senior high school, technical secondary school and technical school, 3 = junior college, 4 = bachelor, 5 = master and above). The length of residence is the year of living in a residential district.

#### 3.3.5. Statistical Analysis

Structural equation modeling (SEM) combined with factor analysis and path analysis is often employed in quantitative research of multi-variable interaction and group comparison because of its advantages. In this study, the SEM method was instrumental in revealing the complicated logic relationship between the life quality, behavior, and residential environment of older adults belonging to different age groups. In this paper, the maximum likelihood estimation method was employed, in addition to the test of mediating effect, referred to the method of Mackinnon et al. [37].

A multiple-factor confirmatory analysis was utilized in the measurement models of leisure environment, landscape environment, neighborhood interaction, outdoor exercise and life quality. It was observed that the factor loads of three observation variables, including maintenance degree, acoustic environment (in the landscape environment measurement model) and religious belief (in the quality of life measurement model), were below standard (0.6). Following the deletion of these three observation variables, the multiple-factor confirmatory analysis was performed once again. Finally, the factor load of all the observed variables was greater than 0.6 [38], and all the measured models had good reliability and validity. The correlation coefficient of landscape environment and leisure environment was 0.627. Therefore, the landscape environment and the leisure environment together constitute the second-order model of the residential environment. 

The model fitting results showed that the chi-square freedom ratio (X2/DF), IFI and CFI did not meet the ideal standard. After model optimization (establishment of the co-variation of the residuals of e 1 and e 2; e 5 and e 6; e 6 and e 7; e 15 and e 16; e 16 and e 17; e 27 and e 28), the final fitness indexes (X2/DF < 5, GFI > 0.90, AGFI P > 0.90, CFI P > 0.9, RMSER < 0.08) met the criteria thereby illustrating the good fit of the model, see Table 2 for details.

## 4. Results

This section may be divided by subheadings. It should provide a concise and precise description of the experimental results, their interpretation as well as the experimental conclusions that can be drawn.

### 4.1. Sample Characteristics

The descriptive statistics of the variables in Table 1 indicate that the life quality of the low-age group and the middle-aged group was similar, but the life quality of the high-age group was significantly lower. The evaluation of leisure environment and landscape environment was good overall, and the differences among the older adults belonging to the three age groups were not obvious. The frequency and duration of exercise decreased with the increase in age and neighborhood interaction decreased with the increase in age. Especially, neighborhood interaction in the high-age group was significantly lower compared with the low-age and middle-age groups. The length of residence in the control variable increased with age. The general average length of residence of older adults was over 20 years, which means the respondents generally lived in a very familiar physical and social environment. Meanwhile, similar characteristics were identified for the levels of income and education. In other words, of the three age groups, respondents in the middle-age group had the highest income and level of education while respondents in the high-age group had the lowest.

### 4.2. Analysis Based on the Models of Full Sample

**Figure 4:**
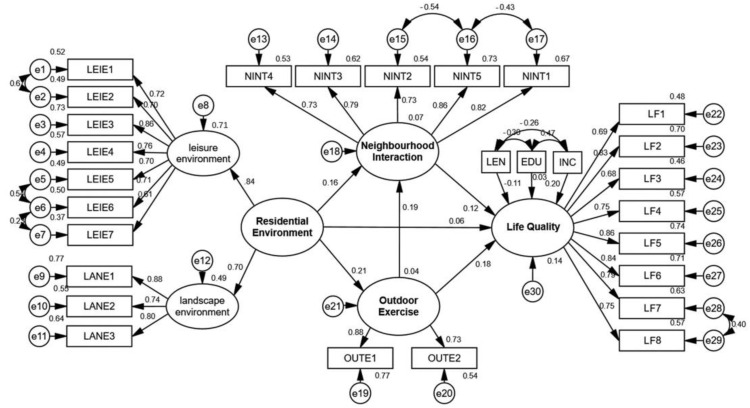
Standardized path diagram for the whole sample model.

The total effects of the residential environment, outdoor exercise and neighborhood interaction on the life quality of older adults were 0.117, 0.200 and 0.124, respectively (See Table 3, Figure 4).

The direct effect of the residential environment on the life quality of older adults was not significant, but the indirect effects clearly indicated that there were complete mediators in the path. Specifically, the effect of the residential environment on the life quality of older adults was completely possible due to the mediating effects of outdoor exercise and neighborhood interaction, in which the mediating effect of outdoor exercise was 0.038, and that of neighborhood interaction was 0.024. Both direct and indirect effects of outdoor exercise on the life quality of older adults were found to be significant; an indication that neighborhood interaction constituted part of the mediating variables in this path.

### 4.3. Comparison of Model Differences among Different Age Groups

**Figure 5:**
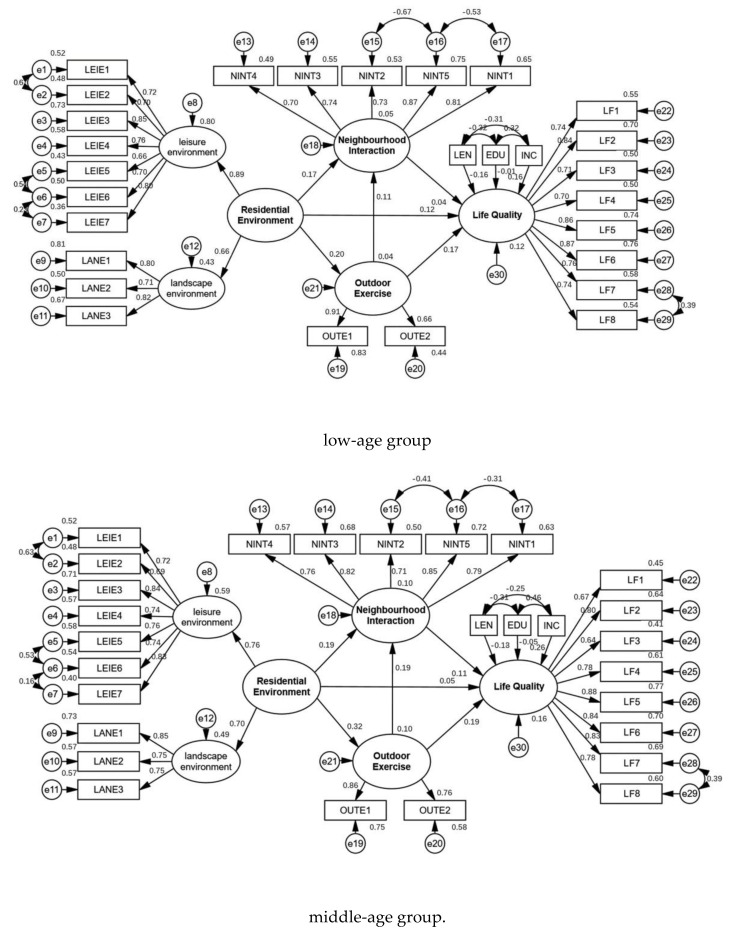

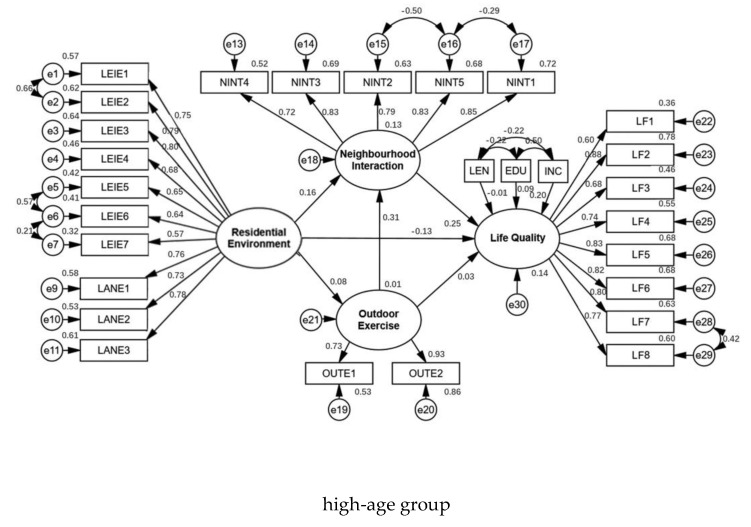
Comparison of models for different age groups.

The data of low-age, middle-age and high-age groups were substituted into the model for group comparison. The fitting result of the high-age group model showed that the fitting result of the second-order measurement model of the residential environment was faulty, and the factor loading of the landscape environment exceeded one. Therefore, the high-age group model was modified to integrate the measurement model of the landscape environment and leisure environment into one variable, and thereafter, the model was re-fitted. This resulted in an adequately fitting result, as presented in Figure 5.

The group comparison result showed that the path coefficient was set to the same *p* < 0.05, which indicated that there were significant differences among different age groups. A comparison of the model paths of older adults in different age groups is presented in Table 4.

In the low-age group, residential environment and outdoor exercise had significant positive effects on the life quality of older adults, the total effect values were 0.159 *** and 0.179 ***, respectively. Meanwhile, neighborhood interaction did not have a significant positive effect. Both direct and indirect effects of residential environment on the life quality of older adults were significant, indicating that there was a partial intermediary variable in the path. Due to the neighborhood interaction having no significant effect on the life quality of older adults, outdoor exercise is an intermediary variable of the living environment. Furthermore, the direct effect of outdoor exercise on the life quality of older adults was significant, but the indirect effect was not significant, indicating that there was no mediation effect in this path. 

In the middle-age group, the life quality of older adults was positively affected by the residential environment, outdoor exercise and neighborhood interaction, and the effect values from high to low were outdoor exercise (0.212 ***), residential environment (0.140 ***) and neighborhood interaction (0.109 *). The direct effect of residential environment on the life quality of older adults was not significant, but the indirect effect was significant. This indicates that outdoor exercise and neighborhood interaction were the complete mediator variables in this path. In which, the mediating effect of outdoor exercise was 0.061, and the mediating effect of neighborhood interaction was 0.027. Meanwhile, the direct and indirect effects of outdoor exercise on the life quality of older adults were significant, indicating that neighborhood interaction was part of the mediator variable in the path. Furthermore, only the direct effect of neighborhood interaction on the on the life quality of older adults was significant.

In the high-age group, only outdoor exercise and neighborhood interaction had significant positive effects on the life quality of older adults, and the total effects were 0.106** and 0.250 **, respectively. In which, the direct effect of outdoor exercise on the life quality of older adults was not significant, but that of indirect effect was significant. The results indicate that neighborhood interaction was the complete intermediate variable in this path. Meanwhile, there was only direct effects of neighborhood interaction on the life quality of older adults.

## 5. Discussion

The main significance of this study lies in the promotion of active aging and the construction of an age-friendly community. Based on the social–ecological model of older adults, the purpose of this study was to explore the relationship among the residential environment, outdoor exercise, neighborhood interactions and the life quality of older adults, and to compare the differences of the model paths among older adults of different age groups. 

Our study confirmed the conclusion of other scholars, that residential environment has a significant positive impact on the life quality of older adults [9,10,11,12,13,14]. Furthermore, we confirmed the existence of the social–ecological model of “residential environment–behavior–life quality of older adults”. The residential environment affects the life quality of older adults by influencing their behavior, including neighborhood interactions and outdoor exercise. In other words, the life quality of older adults may not improve if the improvement in the residential environment fails to increase the intensity of outdoor exercise and neighborhood interactions. Therefore, we believe that a residential environment that can promote the activities of older adults is of great significance to their quality of life. By optimizing the environmental quality of the residential area, improving the design and construction of the aging environment in the residential area, such as arranging sports equipment that is more suitable for older adults to exercise with, providing safe and barrier-free activities places, so as to increase older adults outdoor exercise and neighborhood interaction intensity, etc. This is not only an important measure to improve the life quality of older adults and promote active ageing, but also a key to building ageing-friendly communities. 

Our study confirmed that there are differences in the social–ecological model of older adults in different age groups [19,20,21]. Therefore, in order to build truly ageing-friendly communities and improve the life quality of all older people, targeted advice and strategies based on the characteristics of different age groups are needed. Our study found an interesting rule in the differences of the social–ecological model of older adults in different age groups. That is, with the increase in age, the effect of the residential environment on the life quality of older adults gradually decreased, while that of neighborhood interaction gradually increased. In the low-age group, only the residential environment and outdoor exercise significantly impacted the life quality of older adults. In the middle-age group, the life quality of older adults was significantly impacted by three factors: residential environment, outdoor exercise and neighborhood interaction. As for the high-age group, only outdoor exercise and neighborhood interaction had significant impacts on the life quality of older adults. Furthermore, our study found that while outdoor exercise has a significant positive effect on the life quality of older adults in all age groups, this effect is mediated by neighborhood interactions, and this effect increases with age. Especially for the high-age group older adults, the effect of outdoor exercise on their health is completely mediated by neighborhood interaction. Therefore, neighborhood interaction plays a very important role in the life quality of high-age group older adults. This importance is reflected in two aspects, one is that neighborhood interaction has a higher effect value on the life quality of older adults. The other is that neighborhood interaction plays an important mediating role in outdoor exercise, affecting the life quality path of older adults. Therefore, it is necessary to vigorously create a more friendly community atmosphere, organize health consultation meetings and other themed activities to encourage and help older adults over 80 to participate in neighborhood interaction actively. This will effectively help improve their quality of life. 

The research conclusions of this article confirmed the important role of the residential environment on the behavior and life quality of older adults, and provided a new way of thinking for the government to solve the problem of providing for the aging community. Additionally, this study provides new references for the improvement of public policies for older adults. Governments of various countries, especially the Chinese government, should not only focus on issues such as older adults care services, medical care, and insurance, but also pay attention to the building of a friendly residential environment for older adults. The optimization of the residential environment and the cultivation of an interactive neighborhood atmosphere should also be given importance in government work.

However, there are some shortcomings in the study. First, the size of the survey area, the sample of the communities and older adults are limited. We only selected Xinhua Street in Changning District, Shanghai, for an in-depth survey. However, more data are needed to verify the social–ecological relationships within the residential environment, individual behavior and life quality of older adults. Furthermore, our study is a cross-sectional study that does not fully explain the causal relationship between residential environment, individual behavior, and life quality of older adults. This needs to be supported by the data of continuous longitudinal studies. In addition, the survey was conducted in 2014, and we are conducting a new round of tracking surveys with a view to obtaining updated data. Finally, the measurement of the community environment in this paper was based on a subjective evaluation. In the follow-up research, the combination of the subjective evaluation system and the objective evaluation system will be more helpful to explore the relationship between the residential environment and the life quality of older adults. 

## 6. Conclusions

The residential environment, individual behavior, and the life quality of older adults represent constituents of a social ecosystem that influence each other. At the same time, the social–ecological model paths of older adults in different age groups are different. Therefore, in order to improve the overall life quality of older adults, it is necessary to put forward targeted advice and strategies based on the distinctive characteristics of various age groups. The design and construction of an age-friendly community are not limited to the beauty of the residential environment and the completeness of its facilities. Targeted design, differentiated management and diverse organization should be applied to meet the needs of different groups of older adults, which will yield improvements in the quality of life for all older adults.

## Figures and Tables

**Table 1:** Description of main variables.

Latent Variables	Observed Variables		Variable Items	Mean (All)	Mean (Low-Age)	Mean (Middle-Age)	Mean (High-Age)
Life quality of older adults	**LQ1:** Life Satisfaction		satisfaction with your life and personal situation	7.74	7.81	7.80	7.39
	**LQ2:** Living Standard		satisfaction with your live standard	7.53	7.56	7.63	7.27
	**LQ3:** Health Status		satisfaction with your health status	6.78	7.08	6.71	6.06
	**LQ4:** Personal Achievement		satisfaction with your personal achievement	6.90	6.95	7.16	6.22
	**LQ5:** Sense of Safety		satisfaction with your sense of safety	7.80	7.89	7.86	7.41
	**LQ6:** Living Security		satisfaction with the security of your future life	7.82	7.85	7.89	7.63
	**LQ7**: Interpersonal Relationship		satisfaction with your relationships with others	7.76	7.84	7.87	7.34
	**LQ8:** Neighborhood Harmony		satisfaction with your Neighborhood	7.81	7.81	7.92	7.55
Interpersonal environment	Leisure Environment	**LEIE1:** Exercise Opportunity	There are many opportunities to exercise in the residential district.	3.00	3.02	2.94	3.05
		**LEIE2:** Sports Facilities	Abundant sports facilities in the residential district.	2.95	2.97	2.89	2.98
		**LEIE3:** Suitable for Walking	Very pleasant walk in the residential district	3.31	3.33	3.28	3.32
		**LEIE4:** Enough Trees	Trees in the residential district provide enough shade.	3.14	3.11	3.11	3.25
		**LEIE5:** Walking Convenience	Many places can be walked from our residential district	3.35	3.70	3.60	3.61
		**LEIE6:** Exercise Attraction	Many people often exercised in the residential district	3.18	3.16	3.17	3.27
		**LEIE 7:** Walking Attraction	Many people walking in the residential district frequently	3.32	3.27	3.32	3.45
	Landscape Environment	**LANE1:** Attraction Degree	The residential district is very attractive	2.92	2.92	2.95	2.68
		**LANE2:** Interest of Architecture	The buildings in the residential district are interesting	2.72	2.72	2.70	2.76
		**LANE 3:** Environmental Cleanliness	lots of garbage and waste in the residential district.	3.61	3.61	3.58	3.65
Neighborhood Interaction	**NINT1:** Help		Frequency of you and neighbors helping each other	2.37	2.45	2.38	2.13
	**NINT2:** Care		Frequency of helping your neighbors look after house or property	1.91	1.96	1.91	1.77
	**NINT3:** Communication		Frequency of communication with your neighbor	2.34	2.51	2.35	2.04
	**NINT4:** Activities		Frequency of meeting with neighbors or participating in group event	2.15	2.18	2.25	1.87
	**NINT5:** Chat		Frequency of chatting with your neighbor	2.44	2.51	2.49	2.17
Outdoor Exercise	**OUTE1:** Walking Frequency		times of walking per week (more than 10 minutes)	4.20	4.50	3.58	3.44
	**OUTE2:** Walking Duration		Walking duration (minute)	28.6	31.25	28.7	22.5
Control variable	**INC:** Income		What is the per capita monthly household income	3.33	3.30	3.42	3.22
	**EDU:** Education		What is your education level	2.24	2.17	2.51	1.95
	**LEN:** The Length of Residence		How many years have you lived in this residential district	22.14	20.64	21.12	27.97

**Table 2:** Comparison of fit index before and after model optimization.

	GFI	AGFI	IFI	CFI	RMSEA	X^2^/DF
Pre-optimization model	0.841	0.806	0.869	0.869	0.087	12.029
Post-optimization model	0.913	0.902	0.937	0.936	0.061	5.416
Ideal standard	>0.9	>0.9	>0.9	>0.9	<0.08	<5

**Table 3:** Total effect, direct effect and indirect effect of full sample model.

Independent Variable	Intermediary Variables				Dependent Variable		
	Outdoor Exercise	Neighborhood Interaction			Life Quality of Older Adults		
		Total Effect	Direct Effect	Indirect Effect	Total Effect	Direct Effect	Indirect Effect
Residential Environment	0.210 ***	0.199 ***	0.159 ***	0.040 ***	0.117 ***	0.056	0.062 ***
Outdoor Exercise	----	0.190 ***	0.190 ***	----	0.200 ***	0.176 ***	0.024 ***
Neighborhood Interaction	----	----	----	----	0.124 ***	0.124 ***	----

Notes: *** means significant at the 0.01 confidence level, the significance test chooses the percentile 95% confidence interval two-tailed test method.

**Table 4:** comparison of the model paths in different age groups.

Independent Variable		Intermediary Variables				Dependent Variable		
		Outdoor Exercise	Neighborhood Interaction			Life Quality of Older Adults		
			Total Effect	Direct Effect	Indirect Effect	Total Effect	Direct Effect	Indirect Effect
low-age group (60–69)	Residential Environment	0.198 ***	0.189 ***	0.168 ***	0.021 **	0.159 ***	0.117 **	0.042 ***
	Outdoor Exercise	----	0.107 **	0.107 **	----	0.179 ***	0.175 ***	0.004
	Neighborhood Interaction	----	----	----	----	0.039	0.039	----
middle-age group (70–79)	Residential Environment	0.319 ***	0.251 ***	0.189 **	0.062 ***	0.140 **	0.052	0.088 ***
	Outdoor Exercise	----	0.194 ***	0.194 ***	----	0.212 ***	0.191 ***	0.021 **
	Neighborhood Interaction	----	----	----	----	0.109 **	0.109 **	----
high-age group (80 and above)	Residential Environment	0.083	0.182 ***	0.156 **	0.026	−0.035	−0.083	0.048
	Outdoor Exercise	----	0.309 ***	0.309 ***	----	0.106 **	0.029	0.077 ***
	Neighborhood Interaction	----	----	----	----	0.250 ***	0.250 ***	----

Notes: *** means significant at the 0.01 confidence level; ** means significant at the 0.05 confidence level; the significance test chooses the percentile 95% confidence interval two-tailed test method.

## Abbreviations

WHO: World Health Organization; SEM: structural equation modeling; *p*: *p*-value; X2/DF: Chi-Square/Degree of freedom; IFI: incremental fit index; CFI: comparative fit index; GFI: goodness-of-fit index; AGFI: adjusted goodness-of-fit index; RMSEA: Root Mean Square Error of Approximation
